# A collection of transcriptomic and proteomic datasets from sesame in response to salt stress

**DOI:** 10.1016/j.dib.2020.106096

**Published:** 2020-07-31

**Authors:** Yujuan Zhang, Donghua Li, Rong Zhou, Aili Liu, Linhai Wang, Yanxin Zhang, Huihui Gong, Xiurong Zhang, Jun You

**Affiliations:** a Key Laboratory of Biology and Genetic Improvement of Oil Crops, Ministry of Agriculture and Rural Affairs, Oil Crops Research Institute, Chinese Academy of Agricultural Sciences, Xudong 2nd Road, Wuhan 430062, China; b Cotton Research Center, Shandong Academy of Agricultural Sciences, Jinan 250100, China

**Keywords:** Sesame, Salt stress, RNA sequencing, Proteomic, iTRAQ

## Abstract

Soil salinity is a major abiotic factor affecting the growth and development of important crops such as sesame (*Sesamum indicum* L.). To understand the molecular mechanisms of this oilseed crop in response to salt stress, we examined the transcriptome and proteome profiles of two sesame varieties, with contrasting tolerances to salinity. Here, RNA sequencing and quantitative proteomic analyses of 30 samples from salt-tolerant and -sensitive sesame seedlings under salt stress were carried out. These data can be used for differential gene expression and protein accumulation analyses, based on a genetic aberration or phenotypic differences in sesame responses to salt stress. Our dataset provides an extensive resource for understanding the molecular mechanisms underlying the adaptation of sesame to salt stress, and may constitute useful a resource for increasing the tolerance of major crop plants to raised salinity levels in soils.

Specifications Table

**Table:** 

Subject	Agricultural and Biological Sciences
Specific subject area	Plant transcriptomics
Type of data	Table, Image and Figure
How data were acquired	Illumina HiSeqTM 4000 sequencing platform
Data format	Raw and analyzed
Parameters for data collection	30 samples of 14 day old seedlings prepared from WZM3063 and ZZM4028 varieties with contrasting tolerances to salt. Shoot samples were collected at 0 (control), 2, 6, 12, and 24 h after salt treatment for RNA and protein extraction, cDNA library preparation and sequencing, iTRAQ labeling and LC-MS/MS analysis.
Description of data collection	The RNAseq dataset was collected from paired-end sequencing of sesame cDNA libraries using Illumina HiSeq X ten platform with 2 × 150 bp reads. The raw reads were recorded in a FASTQ file. Raw reads were filtered to remove reads containing adapter or reads of low quality, and clean reads were mapped to sesame genome v.1.0 [1]. The iTRAQ dataset were collected using an AB SCIEX nanoLC-MS/MS system (Triple TOF 6600). The unique peptides were mapping the sesame protein database (assembly S_indicum_v1.0) [2].
Data source location	City: Wuhan Country: China
Data accessibility	The RNA-Seq and iTRAQ raw data were deposited in the Sequence Read Archive of NCBI, under accession number SRP186970 and the ProteomeXchange with identifier PXD013013. Direct URL to data: https://trace.ncbi.nlm.nih.gov/Traces/sra/?study=SRP186970; http://proteomecentral.proteomexchange.org/cgi/GetDataset?ID=PXD013013

**Value of the data**

• These RNA-seq and iTRAQ data obtained from the selected 2 sesame varieties which represent the first complete set of transcriptome and proteomic data generated from sesame varieties with contrasting tolerances to salt.
• These datasets permit comparative transcriptomics and proteomics between salt-tolerant and salt-sensitive sesame varieties. Differential gene and protein expression profiles between varieties could help in understanding the salinity response and tolerance mechanisms of sesame, which helps plant breeders develop traditional breeding and biotechnological approaches to improve stress resistance in sesame.
• These datasets will be of value for future characterization of functional genes and proteins involved in salt stress responses in sesame.
• These datasets are also expected to provide valuable information for the study of molecular mechanisms underlying salt tolerance in other plants.

## 1. Data description

**Fig. 1:**
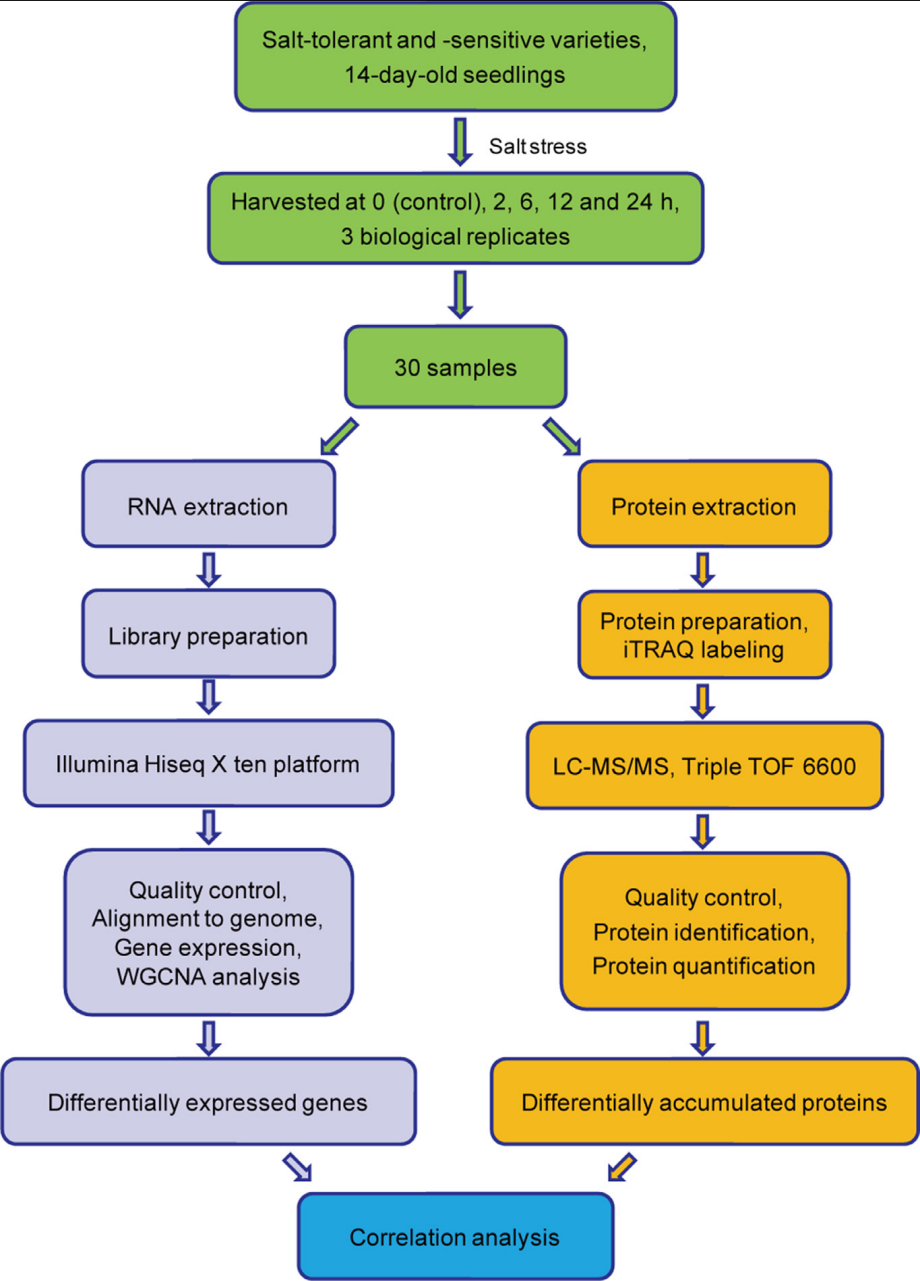
Overview of the study design.

**Fig. 2:**
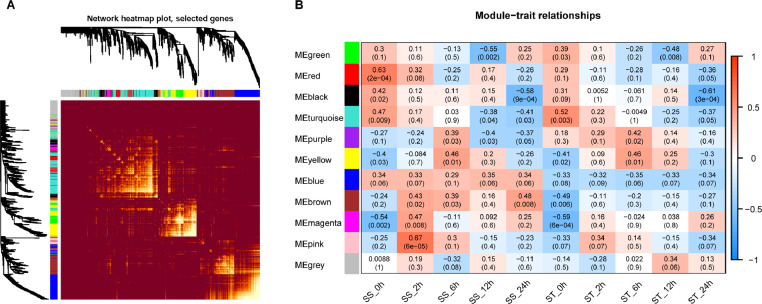
Topological overlap matrix plot of RNA-seq data (A) and plot of module-sample correlation (B).

**Fig. 3:**
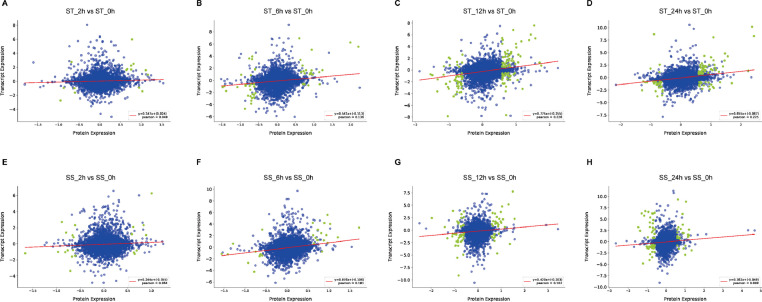
Correlation analysis between mRNA and protein expression levels for differentially expressed genes (proteins) at different salt stress time points.

This dataset aims to provide the transcriptomic and proteomic profiling of 30 samples, from salt-tolerant and salt-sensitive sesame varieties. Fig. 1 provides an overview of our study design. In this work, 30 RNA libraries were sequenced using the Illumina HiSeq X ten platform and 150 bp paired-end reads were generated. Approximately 55 million RNA-seq reads were generated in each sample. After filtering, clean reads were mapped to the sesame genome v.1.0, resulting in 26,620 genes. Using weighted gene co-expression network analysis (WGCNA), 11 co-expression gene modules involved in responses to salt stress were identified in sesame (Fig. 2A and B). At the same time, 30 protein samples, labeled with iTRAQ tags, were analytically separated using an AB SCIEX nanoLC-MS/MS system (Triple TOF 6600). In total, 405,606 spectra and 16,921 unique peptides were generated and 6771 protein species were identified after mapping the sesame protein database (assembly S_indicum_v1.0). Finally, the relationship between mRNA and protein expression levels of differentially expressed genes (proteins), at different salt stress time points, were analyzed (Fig. 3). Stringent technical design at each experimental stage enabled the generation of high-quality RNA-seq and iTRAQ data sets which will be of value for future characterization of genes and proteins expressed in sesame during salt stress responses. These datasets are also expected to provide valuable information for the study of molecular mechanisms underlying salt tolerance in other plants.

## 2 Experimental design, materials and methods

### 2.1 Plant materials and sample selection

The seeds of two sesame varieties were sown and germinated in a box containing half-strength Hoagland solution. The whole cultivation process was accomplished in a growth chamber with a 16/8 h light/dark cycle at 28 °C [1]. 14 day old seedlings of salt-tolerant WZM3063 (ST) and salt-sensitive ZZM4028 (SS) varieties were used for this study. Plants were subjected to salt treatment (150 mM NaCl) at different time points. We collected shoot samples at 0 (control), 2, 6, 12, and 24 h after salt treatment, for RNA and protein extraction. These samples, containing three independent biological replicates, were immediately frozen in liquid nitrogen and stored at −80 °C until use.

### 2.2 RNA extraction, library preparation and sequencing

For each sample, an EASYspin Plus kit (Aidlab, Beijing, China) was used to extract RNA following manufacturer's recommendations. The RNA concentration was measured using a Qubit^Ⓡ^ RNA Assay Kit and Qubit^Ⓡ^ 2.0 Fluorometer (Life Technologies, CA, USA) and the RNA integrity number (RIN) was assessed using the RNA Nano 6000 Assay Kit for the Bioanalyzer 2100 system (Agilent Technologies, CA, USA). RNA libraries were prepared using 3 µg RNA per sample, using a NEBNext^Ⓡ^ Ultra™ RNA Library Prep Kit for Illumina^Ⓡ^ (NEB, USA), following manufacturer's instructions. Library preparations were sequenced on an Illumina HiSeq X ten platform at the Novogene Corporation (Beijing, China) and 150 bp paired-end reads were generated, using methods described previously [3].

### 2.3 RNA-seq data analysis

The raw data (Data Citation 1: NCBI Sequence Read Archive SRP186970) were filtered using Fastq clean v2.0, and clean reads were obtained by removing low quality reads and those containing adapter or ploy-N reads, according to parameters previously reported [4]. At the same time, the Q20, Q30 and GC contents of the clean data were calculated; all downstream analyses were based on these clean, high-quality data. An index of the sesame genome was built using Bowtie v2.2.3 and paired-end clean reads were aligned to the reference genome using TopHat v2.0.12. HTSeq v0.6.1 was used to count the read numbers mapped to each gene, and then the FPKM (fragments per kilobase of transcript per million fragments mapped) for each gene were calculated based on the length of the gene and read count. Correlation analysis of relationships among biological replicates was performed using the software R package (version 3.4.3). The relationship among gene clusters on normalized read counts was analyzed using a WGCNA package (version 1.68) in R [5]. Genes corresponding to the different co-expression modules are listed in Table S1. Differential expression analysis of the two groups was performed using the DESeq R package (version 1.18). Genes with an adjusted *P* value <0.05 were assigned as statistically significant differentially expressed.

### 2.4 Protein extraction, iTRAQ labeling and LC-MS/MS

Protein was extracted from each sample using methods described previously [6]. Protein concentrations were measured using a Bradford assay and protein quality was analyzed on SDS-PAGE. The supernatant from each sample, containing precisely 0.1 mg of protein, was reduced by DTT, underwent iodoacetamide alkylation and was digested with Trypsin Gold (Promega, Madison, WI) at 37 °C for 16 h. After digestion, peptides were applied to a C18 cartridge to remove urea; desalted peptides were then dried by vacuum centrifugation. Desalted peptides were labeled with iTRAQ reagent (iTRAQ^Ⓡ^ Reagent-8PLEX Multiplex Kit, Sigma) following manufacturer's instructions. Differently labeled peptides were mixed equally and then desalted in 100 mg SCX columns. The iTRAQ-labeled peptide mix was fractionated using a C18 column (waters BEHC18 4.6 × 250 mm, 5 µm) on a Rigol L3000 HPLC operating at 1 ml/min and subsequently analyzed on an AB SCIEX nanoLC-MS/MS system (Triple TOF 6600) at Novogene Genetics, Beijing, China.

**Table 1:** Data generated from RNA-sequencing of 30 samples in the NCBI Sequence Read Archive (SRP186970).

Organism	Sample	Replicate	Analysis type	Accession	Accession in SRA
*Sesamum indicum*	ST_0h_1	Biological Replicate 1	RNA-Sequencing (paired)	salt-tolerant	SRX5437947
*Sesamum indicum*	ST_0h_2	Biological Replicate 2	RNA-Sequencing (paired)	salt-tolerant	SRX5437946
*Sesamum indicum*	ST_0h_3	Biological Replicate 3	RNA-Sequencing (paired)	salt-tolerant	SRX5437945
*Sesamum indicum*	ST_2h_1	Biological Replicate 1	RNA-Sequencing (paired)	salt-tolerant	SRX5437944
*Sesamum indicum*	ST_2h_2	Biological Replicate 2	RNA-Sequencing (paired)	salt-tolerant	SRX5437955
*Sesamum indicum*	ST_2h_3	Biological Replicate 3	RNA-Sequencing (paired)	salt-tolerant	SRX5437953
*Sesamum indicum*	ST_6h_1	Biological Replicate 1	RNA-Sequencing (paired)	salt-tolerant	SRX5437954
*Sesamum indicum*	ST_6h_2	Biological Replicate 2	RNA-Sequencing (paired)	salt-tolerant	SRX5437941
*Sesamum indicum*	ST_6h_3	Biological Replicate 3	RNA-Sequencing (paired)	salt-tolerant	SRX5437952
*Sesamum indicum*	ST_12h_1	Biological Replicate 1	RNA-Sequencing (paired)	salt-tolerant	SRX5437942
*Sesamum indicum*	ST_12h_2	Biological Replicate 2	RNA-Sequencing (paired)	salt-tolerant	SRX5437951
*Sesamum indicum*	ST_12h_3	Biological Replicate 3	RNA-Sequencing (paired)	salt-tolerant	SRX5437950
*Sesamum indicum*	ST_24h_1	Biological Replicate 1	RNA-Sequencing (paired)	salt-tolerant	SRX5437949
*Sesamum indicum*	ST_24h_2	Biological Replicate 2	RNA-Sequencing (paired)	salt-tolerant	SRX5437948
*Sesamum indicum*	ST_24h_3	Biological Replicate 3	RNA-Sequencing (paired)	salt-tolerant	SRX5437943
*Sesamum indicum*	SS_0h_1	Biological Replicate 1	RNA-Sequencing (paired)	salt-sensitive	SRX5471706
*Sesamum indicum*	SS_0h_2	Biological Replicate 2	RNA-Sequencing (paired)	salt-sensitive	SRX5471707
*Sesamum indicum*	SS_0h_3	Biological Replicate 3	RNA-Sequencing (paired)	salt-sensitive	SRX5471708
*Sesamum indicum*	SS_2h_1	Biological Replicate 1	RNA-Sequencing (paired)	salt-sensitive	SRX5471709
*Sesamum indicum*	SS_2h_2	Biological Replicate 2	RNA-Sequencing (paired)	salt-sensitive	SRX5471710
*Sesamum indicum*	SS_2h_3	Biological Replicate 3	RNA-Sequencing (paired)	salt-sensitive	SRX5471711
*Sesamum indicum*	SS_6h_1	Biological Replicate 1	RNA-Sequencing (paired)	salt-sensitive	SRX5471712
*Sesamum indicum*	SS_6h_2	Biological Replicate 2	RNA-Sequencing (paired)	salt-sensitive	SRX5471713
*Sesamum indicum*	SS_6h_3	Biological Replicate 3	RNA-Sequencing (paired)	salt-sensitive	SRX5471704
*Sesamum indicum*	SS_12h_1	Biological Replicate 1	RNA-Sequencing (paired)	salt-sensitive	SRX5471705
*Sesamum indicum*	SS_12h_2	Biological Replicate 2	RNA-Sequencing (paired)	salt-sensitive	SRX5471701
*Sesamum indicum*	SS_12h_3	Biological Replicate 3	RNA-Sequencing (paired)	salt-sensitive	SRX5471702
*Sesamum indicum*	SS_24h_1	Biological Replicate 1	RNA-Sequencing (paired)	salt-sensitive	SRX5471699
*Sesamum indicum*	SS_24h_2	Biological Replicate 2	RNA-Sequencing (paired)	salt-sensitive	SRX5471700
*Sesamum indicum*	SS_24h_3	Biological Replicate 3	RNA-Sequencing (paired)	salt-sensitive	SRX5471703

**Table 2.** iTRAQ raw data in ProteomeXchange (PXD013013).

Run groups	Samples	File name	File type	File size
ZMYP_1	ST_0h_1 ST_0h_2 ST_0h_3 SS_0h_1 SS_0h_2 SS_0h_3	20,170,821_ZMYP1.txt.zip	search	2.13M
		20,170,821_ZMYP_1–1.raw	raw	1.58G
		20,170,821_ZMYP_1–10.raw	raw	1.55G
		20,170,821_ZMYP_1–2.raw	raw	1.51G
		20,170,821_ZMYP_1–3.raw	raw	1.66G
		20,170,821_ZMYP_1–4.raw	raw	1.59G
		20,170,821_ZMYP_1–5.raw	raw	1.6G
		20,170,821_ZMYP_1–6.raw	raw	1.43G
		20,170,821_ZMYP_1–7.raw	raw	1.53G
		20,170,821_ZMYP_1–8.raw	raw	1.03G
		20,170,821_ZMYP_1–9.raw	raw	1.51G
ZMYP_2	ST_2h_1 ST_2h_2 ST_2h_3 SS_2h_1 SS_2h_2 SS_2h_3	20,170,815_ZMYP2.txt.zip	search	2.15M
		20,170,815_ZMYP2_1.raw	raw	1.59G
		20,170,815_ZMYP2_10.raw	raw	1.65G
		20,170,815_ZMYP2_2.raw	raw	1.62G
		20,170,815_ZMYP2_3.raw	raw	1.71G
		20,170,815_ZMYP2_4.raw	raw	1.62G
		20,170,815_ZMYP2_5.raw	raw	1.89G
		20,170,815_ZMYP2_6.raw	raw	1.58G
		20,170,815_ZMYP2_7.raw	raw	1.65G
		20,170,815_ZMYP2_8.raw	raw	1.59G
		20,170,815_ZMYP2_9.raw	raw	1.74G
ZMYP_3	ST_6h_1 ST_6h_2 ST_6h_3 SS_6h_1 SS_6h_2 SS_6h_3	20,170,821_ZMYP3.txt.zip	search	2.3M
		20,170,821_ZMYP_3–1.raw	raw	1.59G
		20,170,821_ZMYP_3–10.raw	raw	1.75G
		20,170,821_ZMYP_3–2.raw	raw	1.66G
		20,170,821_ZMYP_3–3.raw	raw	1.15G
		20,170,821_ZMYP_3–4.raw	raw	1.53G
		20,170,821_ZMYP_3–5.raw	raw	1.74G
		20,170,821_ZMYP_3–6.raw	raw	1.76G
		20,170,821_ZMYP_3–7.raw	raw	1.64G
		20,170,821_ZMYP_3–8.raw	raw	1.66G
		20,170,821_ZMYP_3–9.raw	raw	1.63G
ZMYP_4	ST_12h_1 ST_12h_2 ST_12h_3 SS_12h_1 SS_12h_2 SS_12h_3	20,170,821_ZMYP4.txt.zip	search	2.15M
		20,170,821_ZMYP_4–1.raw	raw	1.45G
		20,170,821_ZMYP_4–10.raw	raw	1.58G
		20,170,821_ZMYP_4–2.raw	raw	1.57G
		20,170,821_ZMYP_4–3.raw	raw	1.5G
		20,170,821_ZMYP_4–4.raw	raw	1.57G
		20,170,821_ZMYP_4–5.raw	raw	1.48G
		20,170,821_ZMYP_4–6.raw	raw	1.57G
		20,170,821_ZMYP_4–7.raw	raw	1.53G
		20,170,821_ZMYP_4–8.raw	raw	1.46G
		20,170,821_ZMYP_4–9.raw	raw	783.38M
ZMYP_5	ST_24h_1 ST_24h_2 ST_24h_3 SS_24h_1 SS_24h_2 SS_24h_3	20,170,815_ZMYP5.txt.zip	search	2.27M
		20,170,815_ZMYP5_1.raw	raw	1.46G
		20,170,815_ZMYP5_10.raw	raw	1.55G
		20,170,815_ZMYP5_2.raw	raw	1.59G
		20,170,815_ZMYP5_3.raw	raw	1.56G
		20,170,815_ZMYP5_4.raw	raw	1.58G
		20,170,815_ZMYP5_5.raw	raw	1.54G
		20,170,815_ZMYP5_6.raw	raw	1.66G
		20,170,815_ZMYP5_7.raw	raw	1.55G
		20,170,815_ZMYP5_8.raw	raw	1.67G
		20,170,815_ZMYP5_9.raw	raw	1.63G

### 2.5 iTRAQ data analysis

The raw LC–MS/MS data (Data Citation 2: ProteomeXchange PXD013013) were analyzed using Proteome Discoverer 2.2 software (PD 2.2, Thermo). Search parameters included a mass tolerance of 10 ppm for the precursor ion scans and a mass tolerance of 0.02 Da for the product ion scans. Carbamidomethyl was specified in PD 2.2 as a fixed modification. The oxidation of methionine, acetylation of the N-terminus and iTRAQ 8-plex of tyrosine and lysine were specified in PD 2.2 as variable modifications. A maximum of two mis-cleavage sites were allowed. Protein identification and relative abundance quantitation was carried out based on the sesame genome annotation database (https://www.ncbi.nlm.nih.gov/genome/?term=sesamum) as previously reported [7]. For protein identification, proteins with at least one unique peptide were identified at a false discovery rate of < 1.0% at the peptide and protein levels. Proteins containing similar peptides that could not be distinguished based on MS/MS analysis, were grouped separately. Reporter quantification (iTRAQ 8-plex) was used for iTRAQ quantification as described previously [8]. Protein quantification results were statistically analyzed using the Mann-Whitney Test and significant ratios, defined as *P* value < 0.05 and fold-changes > 1.5 or < 0.67, were used to screen differentially expressed proteins (DEP) [2]. Correlation analysis of biological replicate samples was performed using the IBM SPSS Statistics package version 22 and a heatmap was generated using the Morpheus web server (https://software.broadinstitute.org/morpheus/). Finally, R software version 3.4.3 was used to analyze the relationship between mRNA and protein expression levels of selected genes or proteins.

## 3. Data records

The RNA-Seq and iTRAQ raw data were deposited in the Sequence Read Archive (SRA) of NCBI, under accession number SRP186970 (Data Citation 1) and the ProteomeXchange with identifier PXD013013 (Data Citation 2). Detailed descriptions of the raw data in the SRA and ProteomeXchange are provided in Tables 1 and 2, respectively. In addition, RPKM gene expression and protein relative quantification data of different samples are included in Tables S2 and S3, respectively.

## 4. Technical validation

### 4.1. Quality control of RNA and protein

RIN is positively correlated on uniquely mapped reads in RNA-Seq, and all RNA samples with Agilent Bioanalyzer RIN scores above 6.3 were used to construct RNA libraries. Protein quality was analyzed by SDS-PAGE and all protein samples, used for this study, showed high quality (Fig. S1). Quality values for RNA and protein samples are listed in Tables 3 and 4, respectively.

**Table 3.** RNA sample quality and raw data statistics.

Sample	RNA Quality				RNA-Seq data					
	Total (μg)	RIN	Raw reads	Clean reads	Error rate(%)	Q20(%)	Q30(%)	GC content(%)	Uniquely mapped reads	Uniquely mapped reads(%)
ST_0h_1	3.12	6.5	56,200,078	50,962,020	0.01	97.49	93.89	46.89	46,654,570	91.55
ST_0h_2	5.58	6.7	53,859,392	49,246,562	0.01	97.51	93.71	46.58	44,903,922	91.18
ST_0h_3	12.63	6.7	54,308,450	50,877,488	0.01	97.29	93.48	46.89	46,559,247	91.51
SS_0h_1	5.10	6.7	53,422,124	50,053,352	0.01	97.4	93.7	46.93	44,950,858	89.81
SS_0h_2	4.35	6.5	43,652,426	40,715,530	0.01	97.46	93.8	47.04	36,806,676	90.40
SS_0h_3	8.45	7.2	58,367,766	55,561,642	0.01	97.49	93.68	46.98	51,338,293	92.40
ST_2h_1	6.05	6.4	46,872,818	43,826,216	0.01	97.45	93.45	46.46	38,335,986	87.47
ST_2h_2	6.07	6.3	56,455,556	49,226,762	0.01	97.48	93.63	46.34	44,458,053	90.31
ST_2h_3	4.20	6.4	49,605,272	46,788,354	0.01	97.63	93.99	46.46	42,399,128	90.62
SS_2h_1	4.37	7.7	61,772,050	58,195,088	0.01	97.59	93.87	46.95	52,944,409	90.98
SS_2h_2	6.86	6.6	53,365,476	48,967,336	0.01	97.68	94.07	46.7	45,273,314	92.46
SS_2h_3	4.00	7.9	66,148,362	61,898,474	0.01	97.51	93.71	47.12	56,962,253	92.03
ST_6h_1	6.40	6.3	56,110,052	52,656,078	0.01	97.59	93.86	46.51	48,722,885	92.53
ST_6h_2	4.37	7.1	51,942,334	47,710,022	0.01	97.74	94.19	46.32	43,768,869	91.74
ST_6h_3	6.24	6.3	53,958,372	46,739,214	0.01	98.31	95.66	45.18	41,244,135	88.24
SS_6h_1	6.03	6.4	54,237,112	48,265,958	0.01	97.56	93.83	45.56	43,996,948	91.16
SS_6h_2	5.08	7.3	49,358,766	45,872,538	0.01	97.54	93.77	46.6	42,198,902	91.99
SS_6h_3	6.67	6.7	50,937,298	46,597,730	0.01	97.31	93.39	44.67	41,987,096	90.11
ST_12h_1	5.95	7.4	49,165,946	46,068,752	0.01	97.93	94.74	46.07	42,530,016	92.32
ST_12h_2	7.57	6.5	53,478,642	50,560,280	0.01	97.53	93.78	46.15	46,661,797	92.29
ST_12h_3	7.02	6.5	54,554,576	50,920,208	0.01	97.62	93.92	46.27	47,159,777	92.62
SS_12h_1	5.82	7.1	61,288,330	58,050,112	0.01	97.61	93.93	46.5	53,493,435	92.15
SS_12h_2	7.56	7.4	62,215,438	55,736,000	0.01	97.76	94.09	46.27	51,525,623	92.45
SS_12h_3	11.34	7.2	65,472,130	59,177,334	0.01	97.67	94.04	46.35	54,341,742	91.83
ST_24h_1	5.98	6.9	64,218,272	59,337,384	0.01	97.7	94.11	46.73	54,818,147	92.38
ST_24h_2	6.26	6.5	59,992,120	56,174,124	0.01	97.71	94.13	46.49	50,557,462	90.00
ST_24h_3	16.38	7.8	59,960,160	55,090,912	0.01	97.59	93.85	46.63	50,819,490	92.25
SS_24h_1	6.80	7.6	57,780,738	54,294,106	0.01	97.42	93.51	46.62	49,599,569	91.35
SS_24h_2	6.65	7.5	59,090,286	54,815,124	0.01	97.53	93.72	46.29	50,649,028	92.40
SS_24h_3	7.78	6.3	50,380,854	46,265,926	0.01	97.56	93.8	46.12	42,305,436	91.44

**Table 4.** Protein sample quality and iTRAQ data statistics.

Sample	Protein quality		iTRAQ tags	Run groups	Total spectra	Peptide	Number of protein identifications in each run group	Number of protein identifications in each sample
	Concentration (μg/μl)	Total (μg)						
ST_0h_1	0.61	109.8	113	ZMYP_1	387,760	20,782	4872	4861
ST_0h_2	0.79	142.2	114					4861
ST_0h_3	1.05	189.0	115					4861
SS_0h_1	0.68	122.4	116					4861
SS_0h_2	1.79	322.2	117					4861
SS_0h_3	0.98	176.4	118					4861
ST_2h_1	0.95	171.0	113	ZMYP_2	405,606	20,307	4737	4728
ST_2h_2	1.09	196.2	115					4728
ST_2h_3	0.80	144.0	116					4728
SS_2h_1	1.02	183.6	117					4730
SS_2h_2	2.19	394.2	118					4730
SS_2h_3	1.33	239.4	119					4730
ST_6h_1	1.65	297.0	113	ZMYP_3	394,654	21,506	5006	4996
ST_6h_2	1.56	280.8	114					4996
ST_6h_3	1.09	196.2	116					4996
SS_6h_1	1.33	239.4	117					4996
SS_6h_2	0.76	136.8	118					4996
SS_6h_3	1.84	331.2	119					4996
ST_12h_1	1.28	230.4	113	ZMYP_4	369,499	20,203	4824	4816
ST_12h_2	1.63	293.4	114					4816
ST_12h_3	1.67	300.6	115					4816
SS_12h_1	1.27	228.6	117					4816
SS_12h_2	1.08	194.4	118					4816
SS_12h_3	1.48	266.4	119					4816
ST_24h_1	1.52	273.6	113	ZMYP_5	397,839	21,281	4949	4940
ST_24h_2	1.00	180.0	114					4941
ST_24h_3	2.15	387.0	115					4940
SS_24h_1	1.11	199.8	116					4941
SS_24h_2	2.48	446.4	117					4941
SS_24h_3	0.76	136.8	118					4941

### 4.2. Quality evaluation of RNA-seq and iTRAQ data

The quality of the RNA-seq data was assessed and all samples were deemed of high quality in this study (Table 3). For each sample, over 87.47% of the clean reads with a Q20 rate between 97.29 and 98.31% and Q30 rate between 93.39 and 95.66% were mapped to unique locations in the sesame genome (Table 3). Using correlation analysis of the biological replicates, correlations between the replicates was high (R^2^ > 0.91, Table S4).

**Fig. 4:**
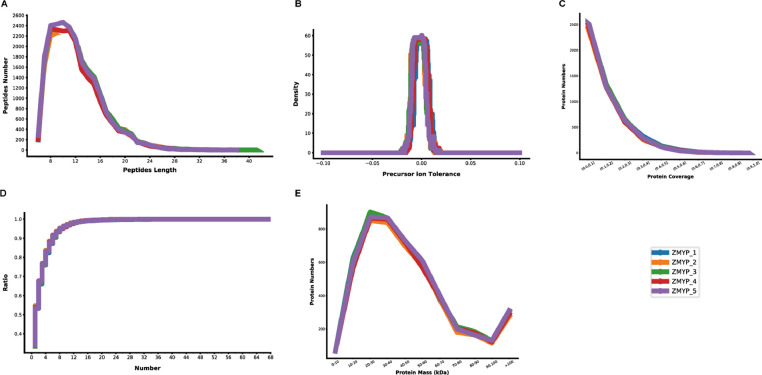
Quality control metrics of iTRAQ data. (A) The length distribution of peptides. (B) Distribution of precursor ion tolerance. (C) Distribution of the unique peptide number. (D) Distribution of protein sequence coverage. (E) Protein mass distribution.

In this study, 30 protein samples, labeled with iTRAQ tags, were divided into five run groups (Table 4). To evaluate the quality of iTRAQ data, the length distribution of peptides, distribution of the precursor ion tolerance, distribution of the unique peptide number, distribution of protein sequence coverage and protein mass distribution for each run group were analyzed (Fig. 4). To evaluate the reliability of protein quantification data, the correlation coefficient of protein expression among 30 samples was measured and a high correlation between biological replicates was recorded (R^2^ > 0.88, Fig. S2).

## 5. Data citations

1 2019. NCBI Sequence Read Archive. SRP186970
2 2019. ProteomeXchange. PXD013013

## Declaration of Competing Interest

Authors declare no conflict of interest.

## Appendix Supplementary materials

Image, application 1

Image, application 2

Image, application 3
