# Supplementary material for: A collection of transcriptomic and proteomic datasets from sesame in response to salt stress
Source: Data Brief. 2020 Jul 31;32:106096. doi: 10.1016/j.dib.2020.106096 (PMC7426533; doi:10.1016/j.dib.2020.106096)
Supplement: Supplementary file 3 [file mmc3.pdf]

A collection of transcriptomic and proteomic data sets from sesame in response to salt stress

Yujuan Zhang[2], Donghua Li[1], Rong Zhou[1], Aili Liu[1], Linhai Wang[1], Yanxin Zhang[1], Huihui Gong[2], Xiurong Zhang[1*], Jun You[1*]

[1] Key Laboratory of Biology and Genetic Improvement of Oil Crops, Ministry of Agriculture and Rural Affairs, Oil Crops Research Institute, Chinese Academy of Agricultural Sciences, Wuhan 430062, China

[2] Cotton Research Center, Shandong Academy of Agricultural Sciences, Jinan 250100, China

* Corresponding authors

E-mail: zhangxr@oilcrops.cn (XZ) and junyou@caas.cn (JY).

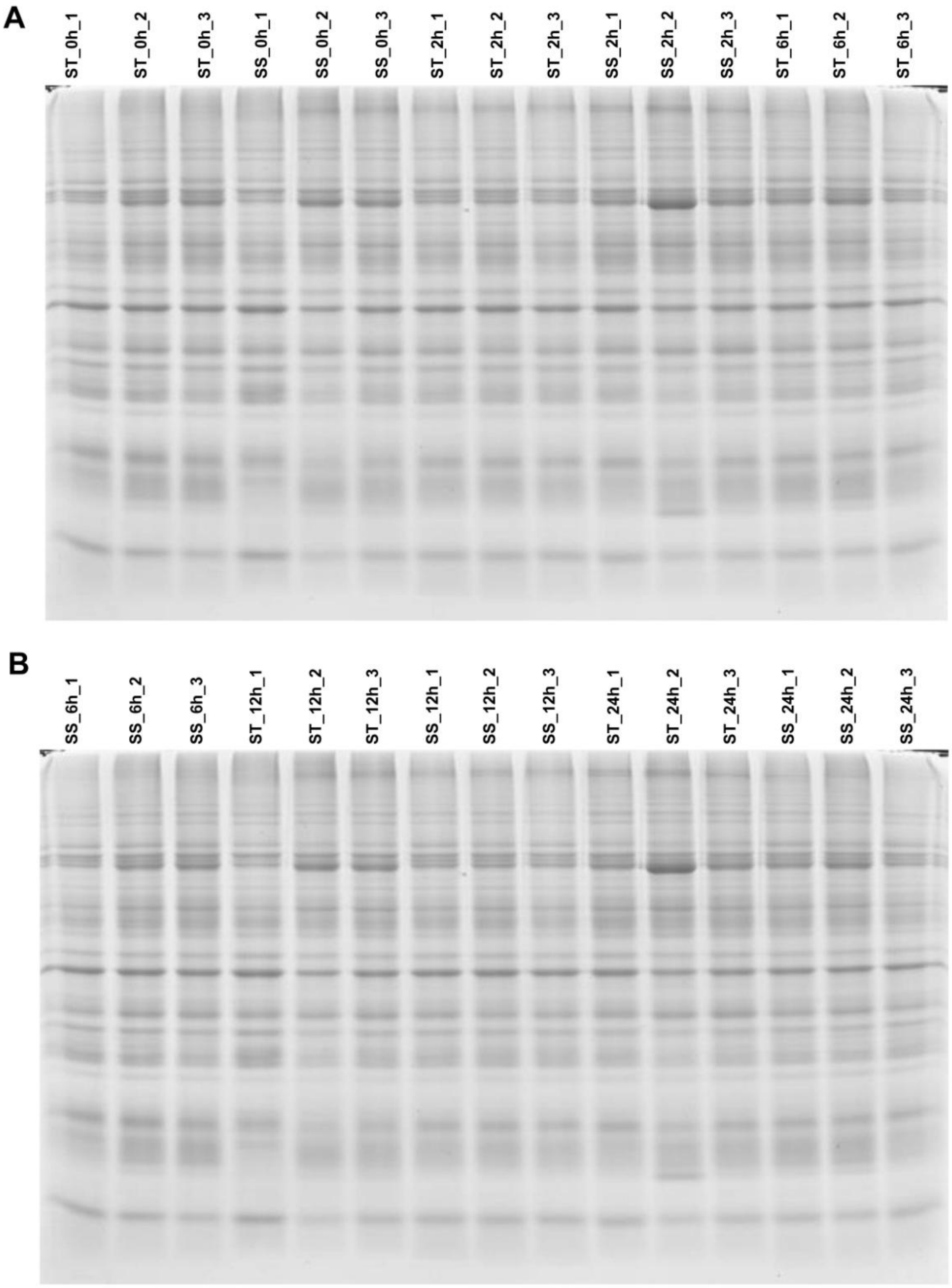

Fig. S1. Protein sample quality evaluation by 12% SDS-PAGE gel. (A) SDS-PAGE gel picture of 15 protein samples. (B) The SDS-PAGE gel picture of other 15 protein samples.

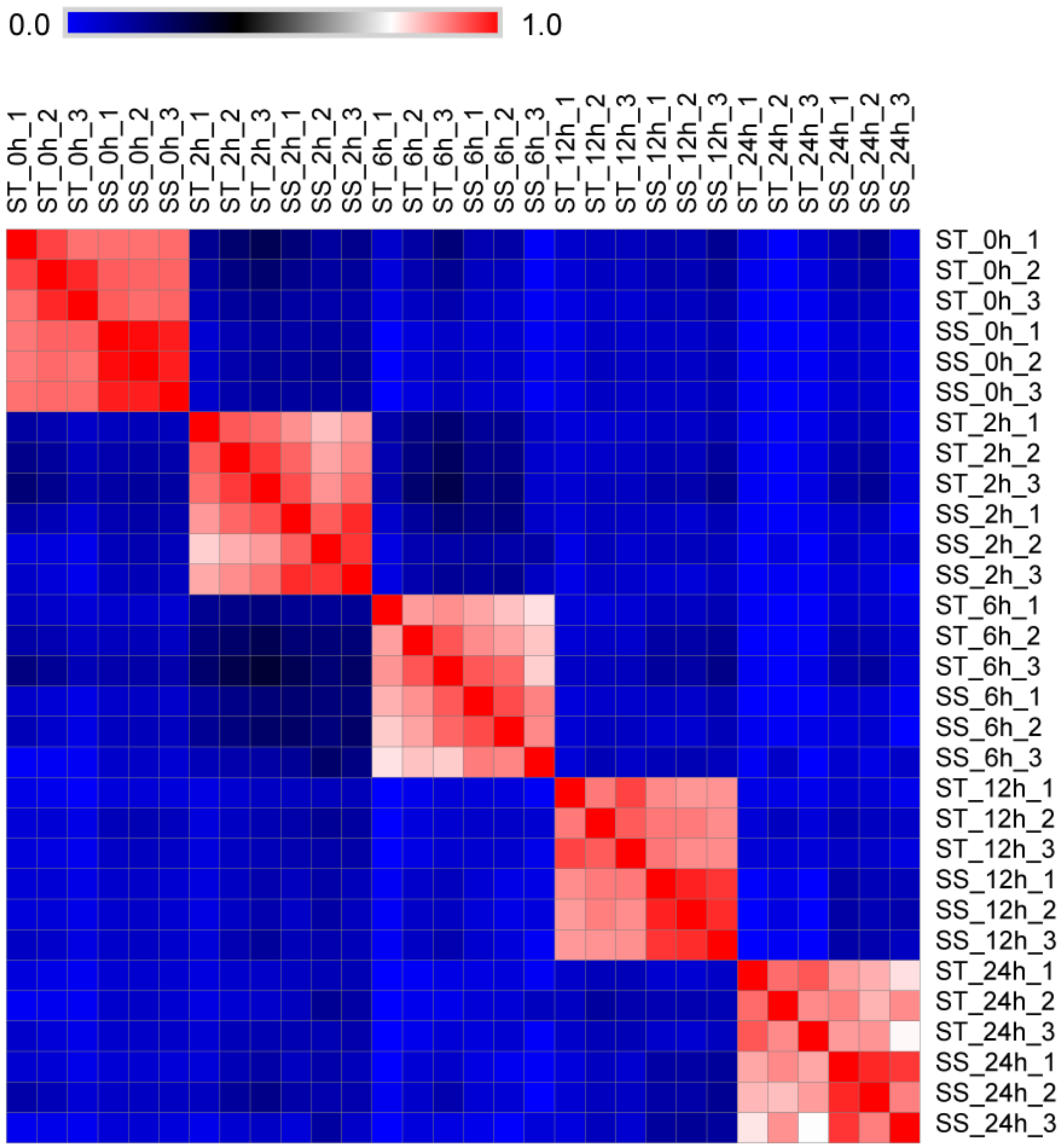

Fig. S2. Correlation analysis of iTRAQ data between biological replicate samples.
